# A Scientific Review of Pesticides: Classification, Toxicity, Health Effects, Sustainability, and Environmental Impact

**DOI:** 10.7759/cureus.67945

**Published:** 2024-08-27

**Authors:** Aishwarya Garud, Satyajeet Pawar, Monika S Patil, Shivani R Kale, Satish Patil

**Affiliations:** 1 Molecular Biology and Genetics, Krishna Institute of Medical Sciences, Krishna Vishwa Vidyapeeth (Deemed to be University), Karad, IND; 2 Microbiology, Krishna Institute of Medical Sciences, Krishna Vishwa Vidyapeeth (Deemed to be University), Karad, IND

**Keywords:** pesticide exposure, agricultural fungicide, local toxicity, insecticides, organophosphates, pesticide poisoning

## Abstract

Over the years, the use of pesticides has risen significantly, primarily to combat undesirable organisms that threaten crucial crops. A variety of pesticides are available, each designed to protect crops from specific threats like fungi, bacteria, and other pests. Other than crop protection, pesticides are also used in controlling insect vector-borne diseases like malaria and Lyme disease in humans. However, the application of these pesticides must be carefully measured and managed to prevent adverse effects on humans, non-target animals, and the environment.

This review delves into the detailed classification of pesticides, exploring their mechanisms of action, applications, benefits, and potential side effects. Understanding the different categories of pesticides, such as herbicides, insecticides, fungicides, rodenticides, and biopesticides, helps us comprehend how each function to control pests. Additionally, we discuss the advantages of pesticide use, including increased crop yields and the prevention of crop diseases, which contribute to food security and agricultural productivity.

This review also addresses the significant concerns related to pesticide usage, such as the development of pest resistance, health risks to humans through exposure and residues in food, as well as the impact on biodiversity and ecosystems. The review emphasizes the importance of using pesticides responsibly and implementing integrated pest management strategies to mitigate this risk of exposure. By providing a comprehensive overview of pesticide classification, mechanisms of action, and implications, this review aims to inform and guide us to the safe and effective use of pesticides for daily agricultural practices.

## Introduction and background

Pesticides are a group of chemicals that are applied to eliminate, repel, or control undesirable organisms. These include insecticides (for insects), herbicides (for weeds), and fungicides (for fungi) [1]. According to the United States Code of Federal Regulations (CFR), a pesticide is any composition or combination of substances formulated to function as a plant regulator, defoliant, or desiccant [2]. Pesticides are vital in agriculture, as farmers employ them to boost crop yields [3]. Based on mode of entry pesticides are classified into systemic, contact, stomach poison, fumigants, and repellents [4]. To fulfill the needs of the growing population and protect crops from insects and pests, farmers are often utilizing pesticides in excess of the recommended dosage, which has been associated with serious health hazards in humans and negative impacts on the environment and wildlife [5].

Organophosphate (OP) pesticides are commonly used to protect crops, but they were also used as nerve agents against soldiers during World War II [6]. While pesticides are essential for safeguarding plants against various infections, they should be strictly administered and used within the recommended dosage. Excessive use of many pesticides leads to severe consequences, such as pesticide poisoning and paralysis, and can even cause death. Furthermore, human exposure to pesticides has been linked to the development of chronic diseases, including heart conditions, cancer, respiratory disorders, and neurological problems [7]. It is crucial to thoroughly evaluate the occupational risks of pesticide exposure and its potential to cause adverse health outcomes.

## Review

There are approximately 385 million instances of unintentional acute pesticide poisoning occurring each year, resulting in around 11,000 fatalities. With a worldwide farming population of around 860 million individuals, roughly 44% of farmers experience pesticide poisoning annually [8]. Every year, nearly 20,000 individuals in developing countries succumb to pesticides through contaminated food [9].

In 2022, global agricultural pesticide use reached 3.70 million tons. Asia was the leading exporter, with 3.5 million tons valued at $21.7 billion. America is a leading pesticide consumer, and the demand increased by 10%, from 1.71 million tons in 2021 to 1.89 million tons in 2022 [10]. In 2022-2023, India produced approximately 299,000 metric tons of pesticides. The Indian chemical industry is highly diverse, encompassing over 80,000 commercial products, and is recognized as a prominent global producer of chemicals [11]. For the year 2022, the consumption of pesticides in India, as reported by the Directorate of Plant Protection, Quarantine and Storage under the Ministry of Agriculture & Farmers Welfare, was 52,466 metric tons [12].

Mounting evidence has suggested that pesticides can act as molecular inducers, manipulating multiple cellular activities in neuroendocrine functions [13]. The primary factors determining the toxicity of pesticides are the pesticide dosage used and the duration of exposure, which together determine whether the toxicity of the pesticide is acute or chronic [14]. Acute toxicity refers to how harmful a pesticide is to animals, plants, and humans when exposed to it for a short time interval. Even a small amount of a highly acutely toxic pesticide can be fatal if it enters the body. In contrast, chronic toxicity assessment involves exposing test animals to the pesticide's active ingredient for an extended duration. Chronic effects result from repeated, low-dose exposures over time and can include adverse health outcomes such as birth defects, fetal toxicity, tumor development, genetic alterations, blood disorders, nerve disorders, endocrine system disruptions, and impacts on reproduction. Assessing chronic toxicity is a more challenging process that entails thorough and lengthy laboratory analysis [14].

India has a total of 256 registered pesticide products, which are classified into two groups: technical grade and formulated. Formulated pesticides are created by incorporating emulsifiers or other agents into the technical grade materials [15]. These pesticides, which include Acephate, Atrazine, Benomyl, Bifenthrin, Captan, Cypermethrin, Dichlorvos, Diclofop-Methyl, Dicofol, Mancozeb, Methomyl, Metolachlor, Oxadiazon, Oxyflourfen, Permethrin, Phosphamidon, Propoxur, Thiophanate Methyl, Triadimefon, and Trifluralin, have been classified by the United States Environmental Protection Agency as potential carcinogens. These pesticides are designated with 'C' and 'B2', meaning they are carcinogenic to humans and probably carcinogenic to humans, respectively, and are also some of the most commonly used pesticides in India [16].

The World Health Organization focuses on acute toxicity when categorizing pesticides. A measure called the lethal dosage (LD50) classifies pesticides into two main types: acute cutaneous (dermal) toxicity and acute oral (mucositis) toxicity is used by WHO. The categorization of pesticides based on LD50. Acute toxicity is categorized based on the lethal dose (LD50) required to kill 50% of a rat population. For cutaneous (dermal) exposure, substances are deemed extremely toxic if the LD50 is less than 50 mg/kg, highly toxic between 50 and 200 mg/kg, moderately toxic between 200 and 2,000 mg/kg, and slightly toxic over 2,000 mg/kg. Similarly, for oral exposure, substances are extremely toxic if the LD50 is less than 5 mg/kg, highly toxic between 5 and 50 mg/kg, moderately toxic between 50 and 2,000 mg/kg, and slightly toxic over 2,000 mg/kg. These classifications help in assessing the risk and safety levels of various chemicals and substances [17].

In the past, chemicals such as dichlorodiphenyl-trichloroethane (DDT), 1,19-(2,2,2-trichloroethylidene)bis(4-chlorobenzene), and other organochlorine (OC) compounds were considered to have low acute toxicity to mammals [18]. However, their use led to phenomena such as eggshell thinning, and bioaccumulation of OCs in fatty tissues in long-lived organisms were observed, leading to the development of less persistent compounds, such as OP and carbamate pesticides [18]. Although these newer pesticides are less persistent in the environment, they tend to be more acutely toxic than OCs.

The World Health Organization has classified pesticides' toxic effects from class Ia to class III and a category of "active ingredients unlikely to present acute hazard”. Class-I technical-grade pesticides are banned or strictly controlled, but not in developing countries. WHO in 1985, estimated that pesticide poisonings affected approximately 3 million people per year and resulted in 220,000 deaths annually [19]. 

Notably, around 99% of the poisoning incidents occur in developing countries, where training for proper pesticide use is limited and the use of appropriate safety equipment is lacking. Furthermore, pesticides’ aerial application, residues in marker food items, and contaminated drinking water can affect both humans and wildlife. Pesticides can move from the target site due to aerial application, and rainfall can leach them into the soil, contaminating groundwater and nearby water bodies [20].

Benefits and harmful effects of pesticides

Over the years, the usage of pesticides has provided numerous benefits to humanity. Pesticide application on agricultural lands protects crops from pest and insect attacks while preventing unwanted plant growth. The key benefits of pesticides include controlling agricultural pest infections, managing vectors of plant diseases, and combating livestock disease vectors and other nuisance organisms [21]. As a result, the global agricultural output has increased exponentially in recent years, largely due to the usage of pesticides, which has reduced crop losses.

Pesticides other than crop protection are also used to control insect vectors. Insect vector-borne diseases are caused by infectious microbes transmitted to humans through blood-sucking arthropods, such as mosquitoes, ticks, sandflies, and flies. These diseases, including malaria, dengue fever, Lyme disease, and leishmaniasis, pose a significant global health threat and have substantial economic and social impacts. Despite extensive control efforts, such as using synthetic insecticides in insecticide-treated nets (ITNs) and indoor residual spraying (IRS), these diseases remain a major concern. Although insecticides have historically been successful in controlling outbreaks, such as eradicating malaria in certain regions, current strategies often overlook the importance of larval control. Larval control is a critical yet often underutilized strategy in managing insect vector-borne diseases, targeting the early stages of vectors to prevent outbreaks. Integrating larval control with other methods can enhance the effectiveness of disease prevention efforts. While pesticides have played a crucial role in boosting agricultural productivity and controlling insect vector-borne diseases, a more comprehensive approach that includes larval control is essential for sustained effectiveness. Future strategies must balance pest management with environmental and public health considerations to address ongoing challenges [22].

An ideal pesticide should be target-specific, attacking only pest organisms of interest, with no unintended side effects on other exposed organisms. Absolute selectivity to harm the target organism only is challenging to attain, as the majority of pesticides are hazardous to both humans and non-target organisms [23]. This non-targeted effect of pesticides is considered a threat to wildlife, the environment, and humans. If proper application of pesticides is not maintained, then pesticides may contaminate soil, pollute water supplies, affecting wildlife, and the environment [20]. Pesticide residues in food supplies are a concerning and consistent problem nowadays, causing pesticide poisoning in humans. Due to their concerning effects as carcinogens and mutants, pesticides like DDT and ethylene dibromide were banned by the Environmental Protection Agency (EPA) [24]. The Environmental Working Group analyzed 47 fruits and vegetables, of which 12 foods had a high concentration of pesticide, eventually named these foods in a group called “dirty dozen” (which included apples, bell peppers, celery, nectarine, strawberries, grapes, cherries, kale, lettuce, carrot, peach, and pear) [25].

Effect on the Environment

Pesticides after application remain in the environment and pollute soil, water (surface water as well as groundwater), turfs, and other vegetation. In addition to killing insects or weeds, pesticides are toxic to many organisms including birds, fish, beneficial insects, and non-target plants. Insecticides are the most acutely toxic class of pesticides, while herbicides can be hazardous to non-target organisms [26]. Pesticides can harm beneficial microorganisms present in soil, as pesticides may cause metabolic changes in these microorganisms [27,28].

Effects on Wildlife

Widespread usage of pesticides exhibits negative effects on non-target organisms, including plants, animals, beneficial insects, aquatic life, and birds. Research studies based on the effect of pesticides on non-targeted wildlife have diagnostic significance as the obtained information can predict probable mechanisms of toxicity in humans. Developmental effects, neurotoxic effects, acetylcholinesterase (AChE) activity, oxidative stress, behavioral toxicity, endocrine function, genotoxicity, and mutagenic effects are observed as pesticide effects in aquatic organisms [29].

Birds show a loss in body weight, changes in hematology, immune system changes, deformed beak and skeleton, and fluid retention in the heart as direct effects of pesticide exposure, while indirect effects on birds include reduced prey populations and habitat changes [30,31]. Animals and pollinators are also disturbed by pesticide accumulation in the ecosystem [32].

Effects on Humans

Every year, over 150,000 people die from pesticide poisoning. This staggering number highlights the need for increasing safety measures and education about the application of pesticides. Most deaths from pesticide exposure result from self-poisoning through ingestion. However, occupational and accidental exposures can also be fatal, usually due to topical (skin) or inhalation of pesticides [33].

Pesticides can enter the body through the mouth (ingestion), nose (inhalation), or skin (dermal absorption) and can cause a range of health problems [34]. The effects of pesticide exposure include neurological, psychological, immune system, and behavioral dysfunctions with symptoms like tremors, memory loss, mood disorders, genotoxicity, and blood disorders. Pesticides can also cause hormonal imbalance, which can lead to infertility. Long-term exposure to pesticides has been associated with an increased risk of cancer [7]. Long-term exposure to these chemicals may lead to the development of tumors in various organs and tissues and may cause blood disorders. Exposure to pesticides has been shown to have a significant association with several types of cancers, including lung cancer, pancreatic cancer, rectal cancer, colon cancer, bladder cancer, leukemia, hematological malignancies, lymphoma, multiple myeloma, prostate cancer, brain tumors, and skin cancer [35]. Previous research studies conducted in both humans and animals have suggested that oxidative stress which is linked to health problems and many serious diseases is primarily induced by organophosphorus pesticides [36].

The toxicity level is determined based on the amount of pesticide required to cause harm, the mode of entry into the body, and the severity of adverse effects. The chronic toxicity of a pesticide is a major concern when assessing its safety for use. This toxicity is evaluated by exposing animals to the active ingredient over an extended period. Chronic toxicity, which refers to the long-term effects of exposure, is also a crucial aspect to consider. It can lead to cancer development, reproductive disorders, and other chronic health conditions. Chronic exposure to certain pesticides has been suspected to cause various detrimental effects on health [37].

The severity of acute toxicity is classified into four categories: extremely toxic, highly toxic, moderately toxic, and slightly toxic. The classification is grounded on the results of toxicological studies carried out on animals, including rats, mice, and rabbits. These studies help to determine the lethal dose of each pesticide and identify the potential hazards associated with exposure to the chemical [38]. The chronic effects of pesticide exposure can cause the occurrence of birth defects in animals and humans [39].

Pesticides known to potentially interfere with hormonal functions are frequently categorized as endocrine-disrupting chemicals (EDCs). An EDC can be described as an external substance that hinders the production, release, attachment, effectiveness, or removal of the body's native hormones. Endocrine disruptors found in the environment, such as polychlorinated biphenyls (PCBs), DDT, dioxin, and certain pesticides, exhibit estrogenic properties and have anti-androgenic effects. In males, exposure to endocrine-disrupting chemicals can lead to infertility, risk of testicular and prostate cancers, atypical sexual development, changes in the functions of the pituitary and thyroid glands, weakened immune responses, and potential neurobehavioral consequences [40]. Meanwhile, in women, pesticide exposure can lead to various abnormal reproductive outcomes, including reduced fertility, miscarriages, stillbirths, preterm deliveries, underweight newborns, birth defects, ovarian irregularities, and disturbances in hormonal function [41].

Disruption of the hormonal function by pesticides

Disruption of the hormonal function by pesticides leads to the following:

Interference With Hormone Synthesis

A study by Zhang et al. inspects how pesticides like glyphosate, thiacloprid, and imidacloprid alter hormone activity, chiefly focusing on estrogen biosynthesis and signaling. Aromatase, a key enzyme in estrogen production, is inhibited by glyphosate through different mechanisms depending on the concentration. In contrast, imidacloprid and thiacloprid had no effect on aromatase activity but mimicked estrogen by activating estrogen receptors at high concentrations and working as endocrine-disrupting chemicals [42].

A review by Shaw examines how abnormal dopamine metabolism in autism has been influenced by environmental, microbial, and genetic factors. It focuses on the enzyme dopamine-beta-hydroxylase (DBH), which converts dopamine to norepinephrine, and how its activity is altered, contributing to autism symptoms. A strong correlation between the rise in autism incidence and the increased use of glyphosate, which disrupts gut microbiota by reducing beneficial bacteria and promoting pathogenic bacterial species Clostridia. Clostridia produces inhibitors of DBH, leading to excess dopamine and reduced norepinephrine, which are common in autism [43].

Interference With Hormone Storage and Release

Fertility rates have declined significantly in recent years, while adverse reproductive outcomes have increased, partly due to pesticide exposure. Women exposed to pesticides face higher risks of reproductive problems. Persistent organic pollutants, such as OP-based compounds, pyrethroids, triazines, and organochlorine pesticides (OCPs), adversely affect human fertility [44].

Interference With Hormone Transport and Clearance

Endocrine disruptors (EDs), including PCBs, DDT, and certain pesticides, can interfere with hormone transport and clearance by mimicking or inhibiting natural hormones. These chemicals disrupt the endocrine system's regulatory functions. Potential impacts of these pesticides include testicular and prostate cancers, abnormal sexual development, undescended testes, chronic inflammation, Sertoli-cell-only syndrome, hypospadias, and altered pituitary and thyroid functions. While the body has mechanisms to regulate hormone levels, the ability to control disruptions caused by low concentrations of EDs, especially in fetuses and young individuals, is uncertain [40].

Binding and Activating the Estrogen Receptor

In a study, 20 pesticides were tested for their ability to activate the estrogen receptor using MCF7 cell proliferation and Yeast Estrogen Screen assays. Fungicides, like fenarimol, triadimenol, and triadimefon, were identified as weak estrogen receptor agonists, causing modest increases in cell proliferation. Fenarimol and dicofol also showed estrogenic activity in yeast cells. Most other pesticides either had no effect or caused negligible responses [45].

The interaction of 49 pesticides with human estrogen receptors (ER-alpha and ER-beta) to understand their potential endocrine-disrupting effects was analyzed. Using stable reporter cell lines (HELN ER- alpha and HELN ER-beta), the pesticides were tested for their ability to activate or inhibit ER-mediated transcription. Fifteen pesticides, including DDT, chlordane, fenvalerate, and toxaphene, were found to agonize ER-alpha in a dose-dependent manner, and some also activated ER-beta. Antagonistic activities were observed for three pesticides on ER-alpha and seven on ER-beta, with chlordecone and methoxychlor being notable antagonists for ER-beta and agonists for ER- alpha [46].

Binding Other Receptors

The effects of pesticides vinclozolin, its metabolite M2, and prochloraz on the rapid androgen signaling via the membrane androgen receptor “ZIP9” in prostate cancer cells were studied by Thomas et al. Known antagonists of nuclear androgen receptors, these pesticides were found to significantly displace testosterone from binding to ZIP9 at low concentrations (1 µM and 10 µM). Additionally, co-treatment with these pesticides blocked or reduced testosterone-induced apoptosis, zinc influx, and Bax gene expression, with prochloraz also attenuating MAPkinase activation. The findings suggest that these pesticides can disrupt ZIP9-mediated androgen signaling, posing potential health risks by interfering with both nuclear and rapid membrane androgen functions, highlighting the broader implications of endocrine disruptor exposure on human health [47].

Various EDCs like pesticides, phthalates, dioxins, and phytoestrogens affect Leydig cells, which produce androgens (male hormones). EDCs can disrupt androgen production, causing reproductive issues in humans and animals. Bisphenol A (BPA) and phytoestrogens can lower testosterone levels, leading to underdeveloped male reproductive organs. Other EDCs, like TCDD, DDE, vinclozolin, linuron, and procymidone, also interfere with Leydig cell function, affecting both fetal and adult male reproductive health. These disruptions can lead to fertility problems and developmental abnormalities [48].

Interference With Hormone Post-receptor Activation

Recent research investigated how 2,3,7,8-tetrachlorodibenzo-p-dioxin (TCDD), a highly toxic dioxin, impacts granulosa cells in pigs when the AhR (aryl hydrocarbon receptor) protein is knocked down. Granulosa cells are crucial for reproductive function. TCDD treatment led to significant changes in the expression of long noncoding RNAs (lncRNAs) in AhR-deficient cells, identifying 57 differentially expressed lncRNAs (DELs) within 24 hours, primarily after just three hours of exposure. This rapid response suggests a quick cellular mechanism against TCDD. Notably, the AhR-deficient cells showed more DELs linked to immune response and transcription regulation than intact cells [49].

Researchers at Peking University developed an improved estrogen-responsive reporter mouse model to study the tissue-specific effects of 2,3,7,8-tetrachlorodibenzo-p-dioxin (TCDD). Using this model, they found that TCDD inhibits estrogen signaling in the liver and kidney but enhances it in the pituitary gland within the same animal. These dual effects occur independently of the expression patterns of related receptors [50].

Interference With the Thyroid Function

Potential increased risks of thyroid cancer have been linked to exposure to endocrine-disrupting compounds like flame retardants, polychlorinated biphenyls, phthalates, and pesticides. This rise in thyroid cancer incidence coincides with greater exposure to these compounds, which can disrupt hormonal signaling and potentially lead to cancers in hormone-sensitive organs like the thyroid. Evidence suggests that certain populations, such as pregnant women and infants, may be particularly vulnerable, with possible transgenerational effects due to epigenetic changes [51].

Interference With the Central Nervous System

Carbamates are pesticides that block the enzyme AChE, causing acetylcholine (ACh) to build up in the nervous system. This can lead to symptoms like sweating, excessive salivation, blurred vision, and in severe cases, paralysis and breathing problems. Unlike other pesticides, carbamates only temporarily block AChE, making them less toxic. However, they can still harm male fertility by disrupting hormone regulation and affecting sperm production [52].

It is important to take precautions when using pesticides to minimize the risk of exposure. This can include wearing protective clothing and equipment, avoiding contact with skin, and ensuring proper ventilation when using pesticides indoors. It is also advisable to limit exposure to pesticides by using alternative pest control methods when possible. Overall, pesticide toxicity is a serious concern that can have significant health impacts. It is important to be aware of the risks associated with pesticide exposure and take appropriate measures.

Types of pesticides

The market offers a wide variety of pesticides, encompassing both inorganic and organic chemicals. These pesticides are categorized as insecticides, herbicides, fungicides, rodenticides, molluscicides, nematicides, and plant growth regulators, further classified based on their primary targets and modes of action [53,54]. Understanding the types and effects of pesticides is crucial to making informed decisions about their usage [54]. By being aware of the potential risks associated with these chemicals, farmers can adopt safer and more sustainable agricultural practices. Farmers need to comprehend the potential impact of each type of pesticide on both crop health and human well-being.

Depending upon the chemical composition, OCs, carbamates, OPs, pyrethroids, and neonicotinoids are considered to be the most common classes of pesticides used in agriculture and pest control. Each of the pesticide classes has its own characteristics and mechanisms of action.

Organochlorines

These were among the earliest synthetic pesticides, but most are now banned due to their persistence in the environment and harmful effects on non-target organisms. DDT is a well-known OC pesticide. It was widely used for insect control but is now banned in many countries as it bioaccumulates in organisms. Lindane (gamma-hexachlorocyclohexane) and endosulfan are said to be the most biodegradable OCs and are still used today [55].

Carbamates

Carbamates are a group of pesticides that inhibit acetylcholinesterase, an enzyme that plays a crucial role in nerve signal transmission. By inhibiting this enzyme, carbamates disrupt the pest’s nervous system, leading to paralysis and death. It affects nerve signaling in non-target organisms [56].

Organophosphates

OPs are another group of pesticides that affect the nervous system. They work by inhibiting acetylcholinesterase, similar to carbamates, causing an accumulation of acetylcholine at nerve synapses and leading to nerve overstimulation and eventually paralysis of the target pests. OPs are a major concern for human health due to their neurotoxic effects and have been linked to increased risk of neurodegenerative diseases like Parkinson's disease [56].

Pyrethroids

Pyrethroids are synthetic chemical compounds known for their effectiveness in controlling a wide range of insect pests. They are widely used in agriculture and pest management due to their potent insecticidal properties. They are modeled after pyrethrins, which are natural insecticides found in chrysanthemum flowers. While pyrethroids are generally less toxic to humans than OPs, they can still pose risks, especially with prolonged exposure. They target the nervous system of insects by affecting sodium channels, causing repetitive nerve impulses and paralysis [57,58].

Neonicotinoids

Neonicotinoids are a relatively newer class of pesticides, are chemically related to nicotine, and are considered highly toxic. They target the nervous system of insects by binding to nicotinic acetylcholine receptors, leading to overstimulation and ultimately death. Neonicotinoids are considered relatively low risk to non-target organisms and the environment and show high target specificity [59].

Each of these pesticide classes has different properties, including their modes of action, persistence in the environment, and potential ecological impacts. It's important to use pesticides carefully and in accordance with regulations to minimize harm to non-target species and the environment.

Furthermore, the classification of pesticides on the basis of the target is as follows:

Fungicides

Fungicides are pesticides used to inhibit the growth and reproduction of fungi, preventing them from spreading and damaging plants. These chemicals work by damaging cell membranes, inactivating critical enzymes or proteins, or interfering with important processes like energy production or respiration in fungi [60]. Examples of common fungicides include Mancozeb, Difenoconazole, Pyraclostrobin, and Azoxystrobin. Some fungi that infect grains produce secondary toxins called mycotoxins, which can cause severe illness or even death in humans and animals. Aflatoxins, ochratoxins, trichothecenes, zearalenone, fumonisins, tremorgenic toxins, and ergot alkaloids are examples of prevalent mycotoxins found in crops [61]. Fungicides are used to reduce mycotoxin contamination, but most developed so far have not been sufficiently effective for managing mycotoxins.

Fungicides can be classified into two types based on their mode of action: single-site fungicides and multisite fungicides. Single-site fungicides target and inhibit a specific site in the fungal metabolic pathway, such as ergosterol biosynthesis, protein biosynthesis, or mitochondrial respiration. In contrast, multisite fungicides act on multiple sites within the fungal metabolic processes [62]. Exposure to various active ingredients in fungicides can lead to different effects on humans. Azoxystrobin, Mancozeb, Maneb, Thiram, and Captan can cause acute symptoms such as irritation to the skin, eyes, and respiratory tract. Chlorothalonil exposure can result in irritation to the skin, mucous membranes of the eyes, and the respiratory tract, as well as allergic contact dermatitis [14].

Fungicides can also be classified based on their chemical composition. Organic fungicides are characterized by the presence of carbon atoms in their molecular structure. Many early fungicides were inorganic, containing sulfur or metal ions like copper, tin, cadmium, and mercury, which are toxic to fungi. Copper and sulfur remain widely used fungicidal agents [63]. Bio-fungicides are another category, comprising naturally derived microbial or biochemical products. Copper compounds like Bordeaux mixture and copper sulfate can also lead to skin, eye, and respiratory tract irritation. Pentachloronitrobenzene can trigger allergic reactions, while sulfur-based fungicides can irritate the skin, eyes, and respiratory tract, diarrhea, and irritated dermatitis in occupationally exposed individuals. Prolonged inhalation of Ziram can cause neural and visual disturbances [14].

Herbicides

Weeds are undesirable, persistent plants that interfere with the growth of desired crops, affecting agricultural land and the country's economy. These plants compete for light, moisture, and nutrients, impacting the quality and quantity of produce. They can also interfere with and damage harvesting equipment and their toxic properties may cause health problems for humans, animals, and the ecosystem. In India, weeds account for the highest potential loss at 37.02% of total agricultural annual losses, followed by insect pests at 27.9% and diseases and other pests at 15.6% [64].

Herbicides also known as weedicides, are chemicals used to control or kill weeds in crop-producing areas. They disrupt the growth of weeds, stopping them from competing with crops for nutrients and water. For many years, chemicals in their crude form, such as oil wastes, rock salts, crushed ores, copper salts, and sulfuric acid have been in use for eradicating weed from car roads and yards [65]. These chemicals are used to destroy all plants and can be categorized as non-selective herbicides. Once treated with these herbicides, the land remains toxic to plants for an extended period. This led to the development of selective herbicides that specifically target and eliminate weeds while leaving desired crops unharmed. Some examples of recent herbicides include Metolachlor, Glyphosate, Atrazine, and 2,4-D (2,4-dichloropnenoxy acetic acid).

Herbicides work by disrupting critical metabolic processes in unwanted plants, such as photosynthesis, hormone production, and cell division. They can be classified as systemic or non-systemic based on how they are translocated within the plant. Herbicides are also categorized by the timing of application (preplant, preemergence, or postemergence) and method of application (soil-applied or foliar). Additionally, they can be selective or non-selective, with multiple potential sites of action [65].

Exposure to chemicals like 2,4-dichlorophenoxyacetic acid and Mecoprop can irritate the skin and mucous membranes, along with symptoms such as vomiting, headache, diarrhea, confusion, aggressive behavior, and muscle weakness, particularly in occupationally exposed individuals. Other active ingredients, including Acetochlor, Atrazine, Dicamba, Mecoprop, Metolachlor, Pendimethalin, and Propanil, can also lead to irritation of the skin, eyes, and respiratory tract. Atrazine exposure may result in abdominal pain, diarrhea, vomiting, irritation of mucous membranes and eyes, and skin reactions. Dicamba can cause loss of appetite, weakness, vomiting, muscle weakness, slowed heart rate, and central nervous system effects. Paraquat exposure can lead to burning in the mouth, throat, chest, and upper abdomen, as well as diarrhea, giddiness, headache, fever, dry and cracked hands, and skin ulceration [14].

Nematicides

Nematodes, which are non-segmented invertebrate microscopic worms, are considered one of the major phytosanitary problems worldwide. These parasitic nematodes are typically 300-1,000 µm in length and 15-35 µm in width. Nematodes attack the root system of crops, making profound changes such as removing photo-assimilates and reducing the plants' absorption capacity of water and nutrients, as well as causing root lesions and yield loss [66,67]. Although the infestation occurs in the root system of the crop, the reflex symptoms are also detected in the shoot.

Nematicides are chemicals used for controlling the microscopic worms that can damage crops. They kill or repel nematodes, preventing them from feeding on plant roots and causing stunted growth and other problems. Common nematicides include carbofuran, fumigants, and OPs. Nematicides applied directly to the soil are classified as fumigants or non-fumigants based on their movement in the soil. Fumigant nematicides are considered biocidal, as they act on fungi, bacteria, seeds, and other soil organisms. Fumigants have high vapor pressures and diffuse rapidly through the soil's porous network in the gas phase. Methyl bromide was the first fumigant nematicide used to control nematodes in crops like tobacco, but its application can cause ecological disturbance and phytotoxicity [67-69].

After the 1940s, strategies were pursued to improve the application technology and reduce the toxicity of nematicides. Less toxic products with nematocidal properties, and without a vapor phase, were developed and termed non-fumigant nematicides. Non-fumigant nematicides are granular or liquid compounds that are water-soluble and have either contact, nematistatic, or systemic activity against nematodes. They represent two main classes of chemical compounds: OPs (terbufos, ethoprophos, fenamiphos) and carbamates (aldicarb, carbofuran, carbosulfan, oxamyl) [69].

Insecticides

Approximately 10,000 species out of the 1 million known insects are considered as crop-harming pests. Of these, around 700 insect species are responsible for most damage to crops in fields and storage facilities worldwide [70]. Insecticides are chemicals that are used to control these insect pests, like aphids, caterpillars, and beetles. Insecticides other than crop protection are also used to control insect vectors, such as mosquitoes and ticks, which are primarily responsible for spreading public health diseases like malaria and Lyme disease.

Insecticides work by either killing directly or by disrupting pests' growth and development. Some examples of common insecticides include pyrethroids, OPs, Malathion, carbaryl, acetamiprid, Thiomethoxam, and neonicotinoids. Different classes of chemicals that are toxic to many other organisms and show strong toxicity to intended insects are used in making insecticides. Most of the insecticides affect non-target organisms similarly as they target the target organisms [71]. Insecticides interrelate with many target and non-target sites of many molecules.

Insecticides are primarily neurotoxic, targeting the nervous systems of both insects and humans. However, they may also impact other organs and bodily systems [72]. Insecticides can have harmful health effects ranging from acute irritation and pain to death in non-target organisms including humans, birds, and wildlife. Depending on the dosage and duration of exposure, insecticides can affect the central nervous system, skeletal muscles, digestive system, cardiovascular system, respiratory system, eyes, reproductive system, endocrine system, and skin, and affect the immune system at the cellular and molecular levels.

Exposure to the OP insecticide Acephate can lead to symptoms including headaches, excessive salivation, tearing, muscle twitching, nausea, diarrhea, respiratory depression, seizures, fatigue, and pinpoint pupils. Insecticide exposure to Aldicarb and Carbaryl may manifest as muscle weakness, dizziness, sweating, headache, increased salivation, nausea, abdominal pain, diarrhea, and, in serious cases, nervous system depression and potentially pulmonary edema. Exposure to Chlorpyrifos, Methyl Parathion, Phosmet, and Malathion can result in similar symptoms. The OC insecticide Endosulfan may cause itching, burning, tingling of the skin, tremors, mental confusion, seizures, respiratory depression, and even coma. Pyrethroids, a synthetic class of insecticides, can cause abnormal facial sensations, dizziness, salivation, headaches, fatigue, vomiting, diarrhea, increased irritability to sounds or touch, seizures, and numbness [14].

Rodenticides

Rodenticides are chemicals used to control rodents such as mice, rats, squirrels, and bats, which can cause damage to crops. These substances typically work by disrupting normal blood clotting, leading to internal bleeding. Most rodenticides cause death by interfering with the functioning of the rodents' nervous system. Some common examples of rodenticides include warfarin, bromadiolone, and zinc phosphide. Commonly employed rodenticides include substances like warfarin, 1080 (sodium fluoroacetate), ANTU (the legal label for alpha-naphthylthiourea), and red squill. Additionally, fumigants like sulfur dioxide, carbon monoxide, hydrogen cyanide, and methyl bromide are also efficient in controlling rodents [73].

Rodenticides, such as phosphorus paste, barium carbonate salt, and powders like zinc phosphide, white arsenic, thallium sulfate, strychnine, strychnine sulfate, and calcium cyanide, are mixed with bait and strategically placed for rodents to ingest. Other than rats, these rodenticides are toxic to non-target organisms like aquatic animals, wildlife, and humans. Most of the rodenticides are highly toxic as they act slowly blocking vitamin K synthesis which is essential for normal blood clotting, leading to uncontrollable bleeding and eventually death [74].

Symptoms like nose, gum, or skin bleeding are seen in rodenticide poisoning in humans, while in some cases internal bleeding can occur due to Anticoagulant rodenticides [75]. Zinc phosphide rodenticide may cause vomiting, chills, shortness of breath, coughing, convulsions, or even coma. Breathing zinc phosphide dust or phosphine gas may also cause anxiousness and difficulty breathing [76].

Biopesticides

Pesticides that are extracted from natural sources, such as bacteria, fungi, minerals, and plant extracts, which exhibit a positive shift in the field of pest management are biopesticides. They are often used as a good alternative to synthetic pesticides, as they are considered to be safer, sustainable, and more eco-friendly solutions for pest management as they, often target specific pests or diseases while minimizing harm to non-target organisms. Some examples of common biopesticides include Bacillus thuringiensis, spinosad, neem oil, ladybugs, genetically resistant crops, and azadirachtin [77].

Semiochemicals are chemical signals used by organisms for communication, including pheromones that can attract or repel pests. These can be employed to disrupt pest behavior, lure them into traps, or deter them from crops [78]. Plant-incorporated protectants are genetically engineered plants that produce their own defense against pests. For example, BT cotton is produced by inserting toxin-producing gene from Bacillus thuringiensis organisms by genetically modifying the crop, enabling them to express insecticidal proteins [79]. It reduces the need for external pesticide applications, making it a more sustainable and cost-effective approach. Many plants and microorganisms produce natural compounds with pesticidal properties. This method involves using natural enemies of pests, such as predators and parasitoids, to manage pest populations. Encouraging the presence of beneficial organisms in agricultural ecosystems helps to control pests without the need for chemical interventions [80]. Advances in genetic research are enabling the development of crop varieties and livestock breeds with natural resistance to pests, reducing the need for external pest management measures.

Promising developments in biopesticide research and development have the potential to reduce the reliance on conventional chemical pesticides and address the challenges of pest resistance, environmental damage, and health concerns linked with pesticide use. The adoption of these sustainable and innovative approaches will likely play a significant role in the future of pest management.

## Conclusions

Pesticides are essential for combating pests and diseases that affect agriculture and human health. They protect crops from harmful pathogens, weeds, and insects, while also safeguarding humans from vector-borne diseases. However, their use requires careful consideration and strict adherence to safety measures due to potential health risks and environmental impacts. Pesticide usage carries significant health risks, including acute effects like skin irritation and respiratory issues, as well as chronic exposure that can lead to serious conditions such as cancer and neurological disorders. Vulnerable populations, including children, pregnant women, and farmworkers, are particularly at risk. Furthermore, pesticide runoff contaminating nearby water supplies, and pesticide residues in food further increase exposure, emphasizing the need for strict safety standards and monitoring.

Integrated pest management offers a sustainable approach by minimizing pesticide use through methods like biological control and crop rotation. Responsible use and exploration of alternative practices, such as organic farming, are crucial to mitigating the negative effects of traditional pesticides. While pesticides are vital tools for managing agricultural challenges, they should be handled with caution. Alternative, more sustainable approaches should be explored whenever possible to strike a balance between protecting crops and the environment.
